# Supplementary material for: Transcription can be sufficient, but is not necessary, to advance replication timing
Source: bioRxiv. 2025 Feb 5:2025.02.04.636516. Preprint. [Version 1] doi: 10.1101/2025.02.04.636516 (PMC11838563; doi:10.1101/2025.02.04.636516)
Supplement: Supplement 1 [file NIHPP2025.02.04.636516v1-supplement-1.pdf]

bioRxiv preprint doi: https://doi.org/10.1101/2025.02.04.636516; this version posted February 5, 2025. The copyright holder for this preprint (which was not certified by peer review) is the author/funder, who has granted bioRxiv a license to display the preprint in perpetuity. It is made available under aCC-BY 4.0 International license.

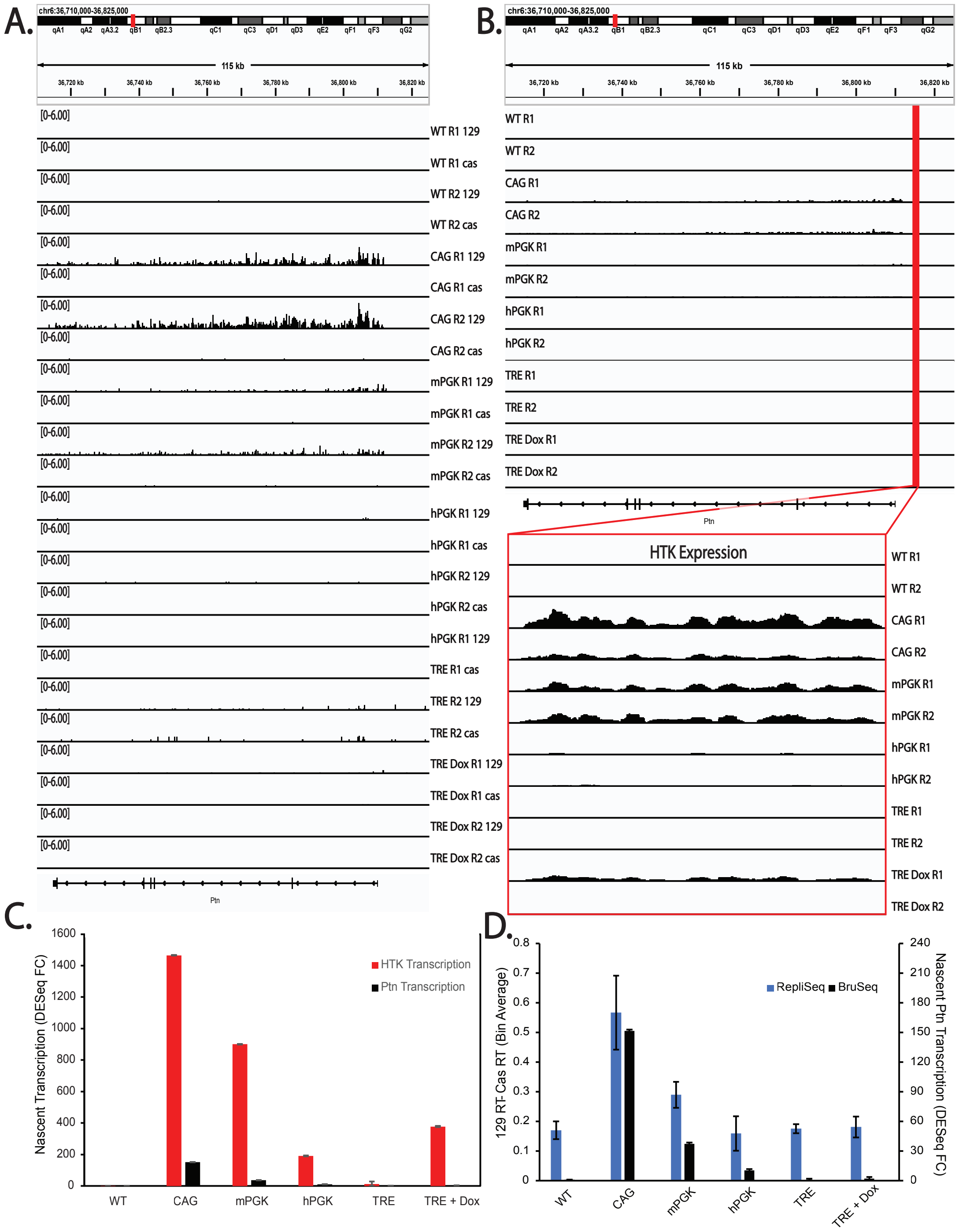

Supplemental Figure 1: A) Genome Browser tracks of genome-parsed Bru-Seq replicates for the cell lines with the Promoter-HTK insertions (vector sequences not included), indicating that Ptn read through reads originate exclusively from the 129 allele. B) Genome Browser tracks of unparsed Bru-Seq replicates for the cell lines with the Promoter-HTK insertions, showing only the inserted allele for each replicate. Top tracks display the read through expression of the Ptn gene, while the zoomed in, bottom tracks display the expression of the HTK gene. C) Bar graph comparing HTK and Ptn read through expression in the Promoter-HTK cell lines. D) Bar graph of the levels of read through Ptn expression in relation to changes in the RT of the Ptn domain.

bioRxiv preprint doi: https://doi.org/10.1101/2025.02.04.636516; this version posted February 5, 2025. The copyright holder for this preprint (which was not certified by peer review) is the author/funder, who has granted bioRxiv a license to display the preprint in perpetuity. It is made available under aCC-BY 4.0 International license.

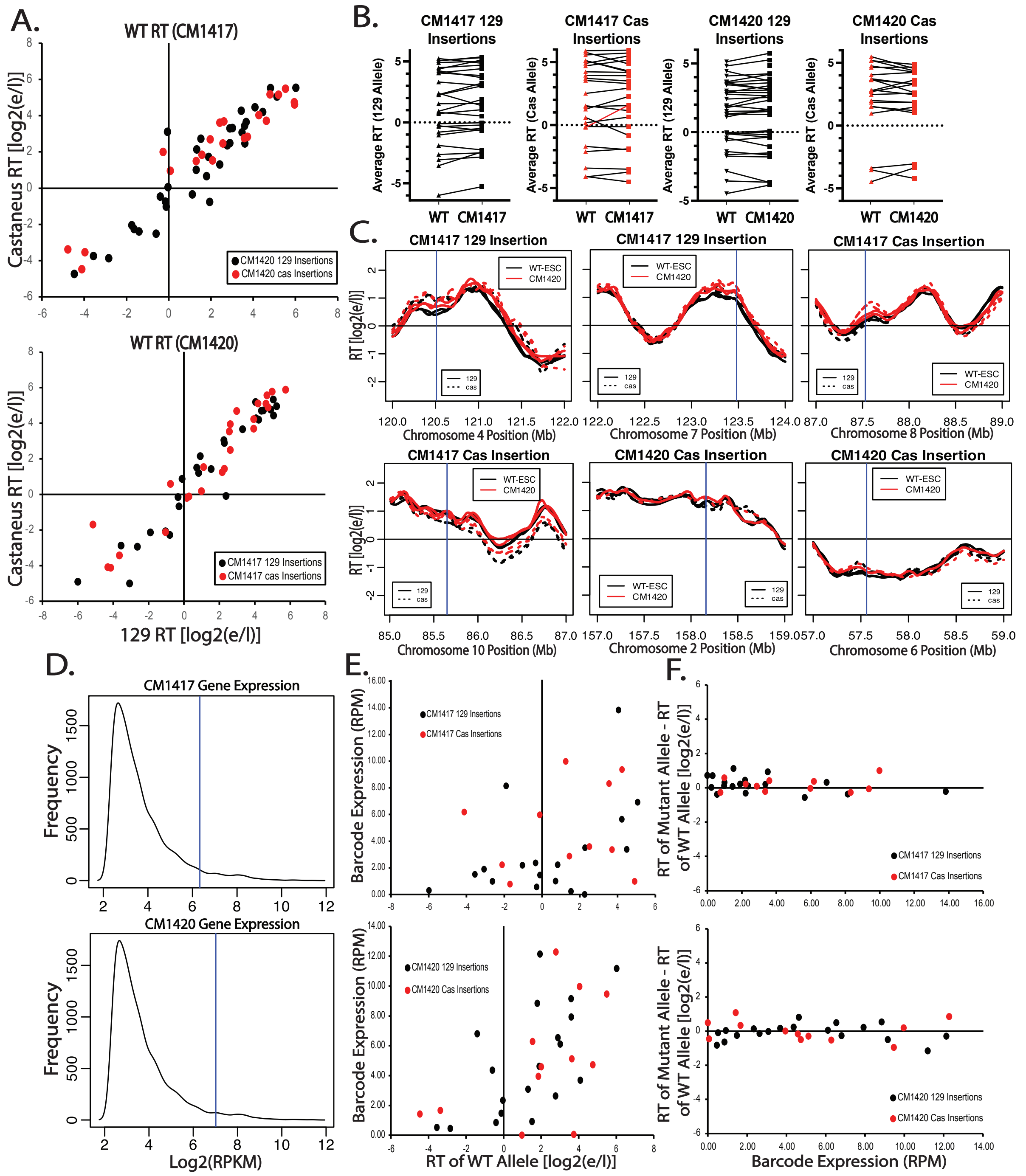

Supplemental Figure 2: A) Scatter plots of the RT (129 vs Cas) of the WT parental alleles of all CM1417 (left) and CM1420 (right) ectopic insertion sites. Black and red dots represent loci where the eventual insertion was made in the 129 and Cas allele respectively. B) RT of the CM1417 129 insertions (left), CM1417 Cas insertions (middle-left), CM1420 129 Insertions (middle-right) and CM1420 Cas Insertions (right) before (WT, triangles) and after (CM1417 or CM1420, squares) the PB insertion. There were no statistically significant advances, calculated using RepliPrint, in RT across both clones, with the exception of the site inserted at Chromosome 8, 87.5Mb, of the Cas allele of CM1417. The locus with the statistically

bioRxiv preprint doi: https://doi.org/10.1101/2025.02.04.636516; this version posted February 5, 2025. The copyright holder for this preprint (which was not certified by peer review) is the author/funder, who has granted bioRxiv a license to display the preprint in perpetuity. It is made available under aCC-BY 4.0 International license.

significant RT change is marked with a red line in the CM1417 plot. C) RT plots of six insertion sites where the largest advances in RT of the insertion allele were observed, including the statistically significant RT change at the Cas Chromosome 8 allele (top right). D) Distribution of all expressed genes in CM1417 (top) and CM1420 (bottom) clones. The x-axis represents the log2(RPKM) expression level of each gene and the y-axis represents the number of genes that are expressed at each level. RPKM was calculated from the average read count for each gene across two replicates. Genes with extremely low or no expression (RPKM<5) were excluded from the plot. Average GFP expression, calculated by dividing the GFP RPKM of each clone by the number of insertions in that clone, is marked with vertical blue lines. E) Position effect of each insertion locus on the expression level of each insertion. To differentiate between expression levels at each insert, only reads containing the 16-nucleotide barcode were extracted from the unprocessed FastQ and were used to calculate the RPM for each barcode. F) Effect of transcription of individual insertions on the RT of the insertion site. The Y-axis range represents the dynamic range of RT in each clone.

bioRxiv preprint doi: https://doi.org/10.1101/2025.02.04.636516; this version posted February 5, 2025. The copyright holder for this preprint (which was not certified by peer review) is the author/funder, who has granted bioRxiv a license to display the preprint in perpetuity. It is made available under aCC-BY 4.0 International license.

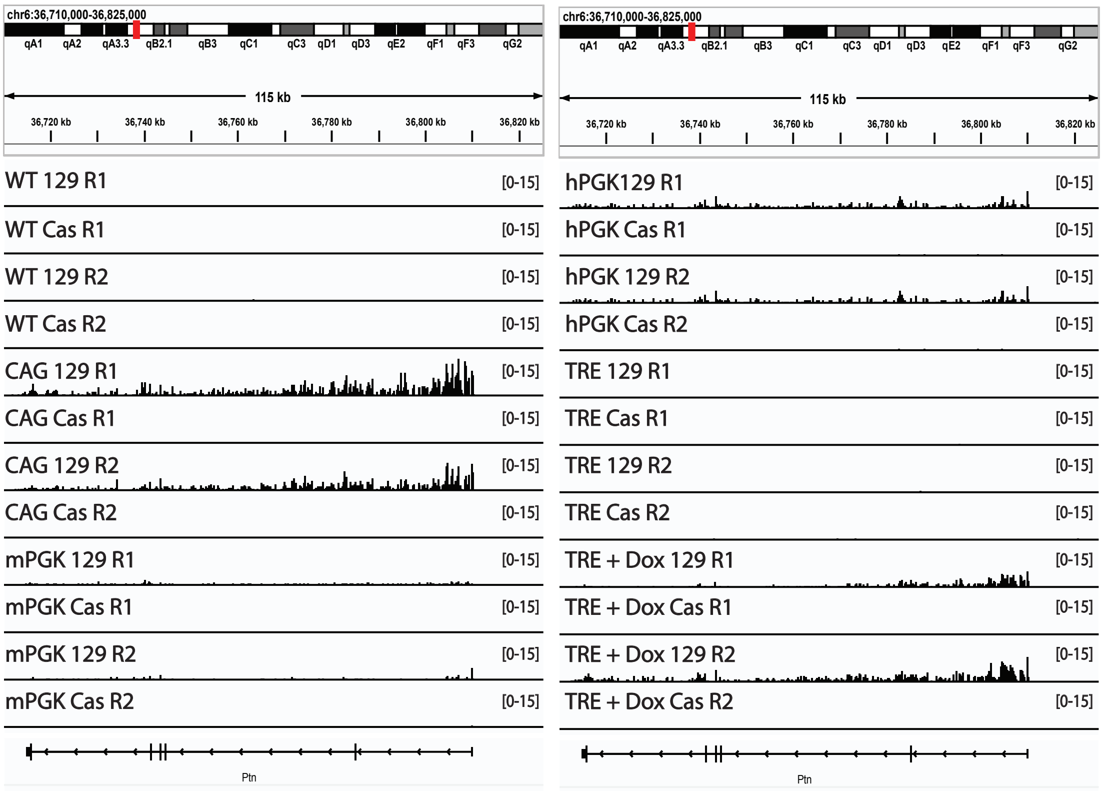

Supplemental Figure 3. Genome Browser track of parsed Bru-Seq replicates for the cell lines in which the inserted promoters drive Ptn transcription.

bioRxiv preprint doi: https://doi.org/10.1101/2025.02.04.636516; this version posted February 5, 2025. The copyright holder for this preprint (which was not certified by peer review) is the author/funder, who has granted bioRxiv a license to display the preprint in perpetuity. It is made available under aCC-BY 4.0 International license.

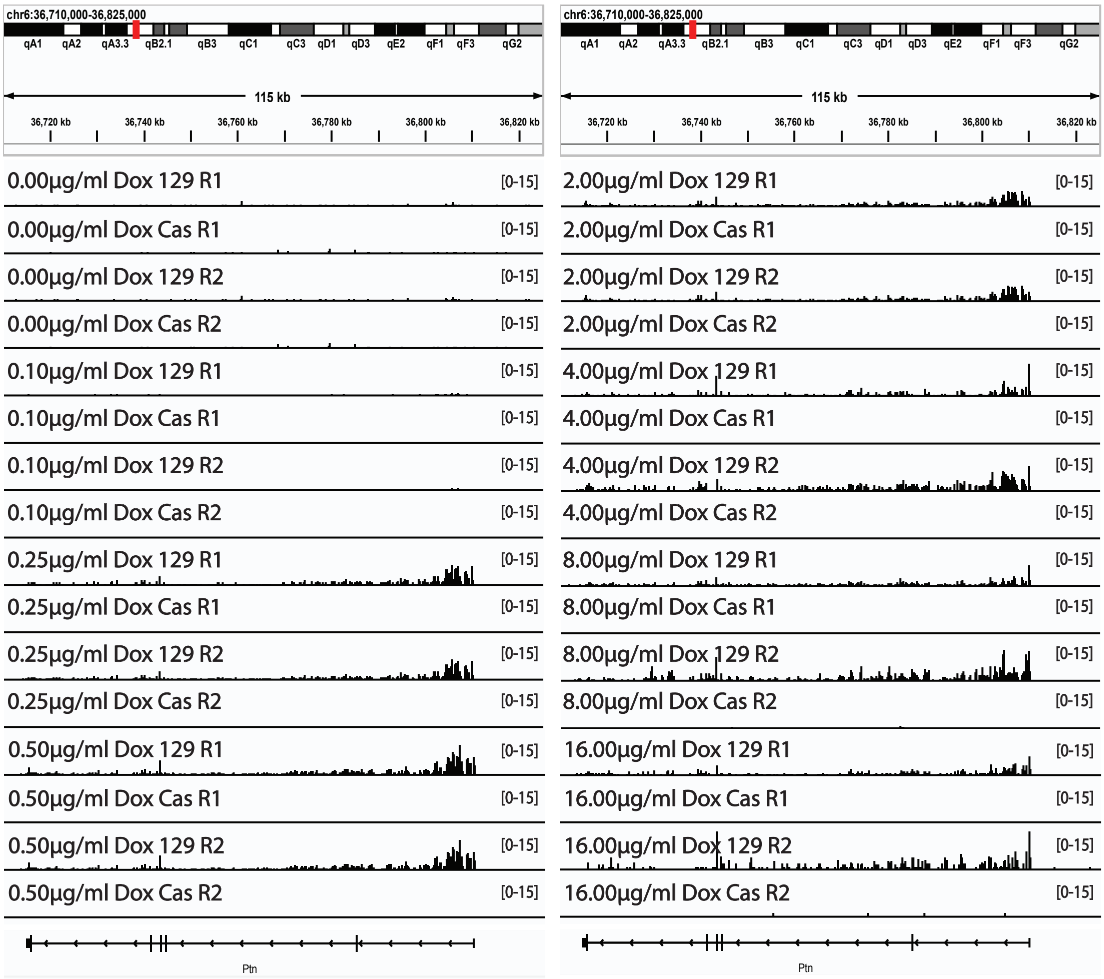

Supplemental Figure 4. Genome Browser track of parsed Bru-Seq replicates displaying Ptn expression at different concentrations of Dox in the TRE-Ptn cell lines.

bioRxiv preprint doi: https://doi.org/10.1101/2025.02.04.636516; this version posted February 5, 2025. The copyright holder for this preprint (which was not certified by peer review) is the author/funder, who has granted bioRxiv a license to display the preprint in perpetuity. It is made available under aCC-BY 4.0 International license.

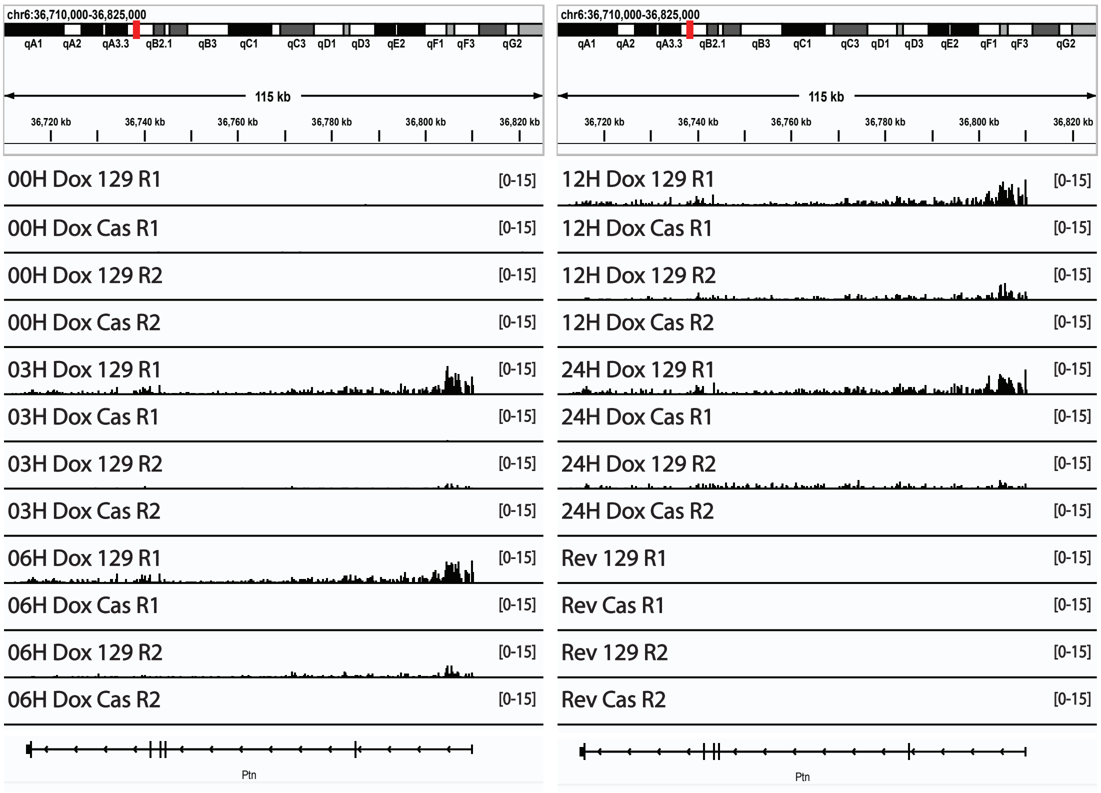

Supplemental Figure 5. Genome Browser track of parsed Bru-Seq replicates displaying Ptn expression at different time points after the addition of Dox in the TRE-Ptn cell lines.
